# Antibody-Drug Conjugates Used in Breast Cancers

**DOI:** 10.1155/2021/9927433

**Published:** 2021-06-28

**Authors:** Aram J. Abbas, Marah F. Ibrahim, Maher S. Saifo

**Affiliations:** ^1^Faculty of Medicine, Damascus University, Damascus, Syria; ^2^Faculty of Pharmacy, Alsham Private University, Damascus, Syria

## Abstract

The prognosis of breast cancer has radically changed in recent years and continues to improve due to the broad application of effective therapies. New targeting strategies including targeted delivery of cytotoxic drugs via receptor-targeting agents have been developed. We summarize recent publications and developments of novel antibody-drug conjugates (ADCs) used to control breast cancer.

## 1. Introduction

Cancer is the second main cause of mortality worldwide [1]. Breast cancer is the most common cancer in women, and the most common cancer overall [2]. A subtype of breast cancer overexpresses HER2 receptors and is called HER2-positive (HER2+); HER2+ breast cancer accounts for 15–20% of all breast cancers and is associated with poor patient outcome and aggressive phenotype [3]. For many years, the therapies of the breast cancer were based on known biomarkers such as estrogen receptor (ER), progesterone receptor (PR), and human epidermal growth factor receptor 2 (HER2) [4–6]. One of these treatments is trastuzumab; a humanized monoclonal antibody that targets HER2 receptor, leading to angiogenesis inhibition, diminished microvessel density, and better overall survival rates in patients with HER2-positive breast cancer [7]. However, resistance to trastuzumab has been reported [8, 9], in addition to several severe adverse effects such as cardiac toxicity [10]. In general, naked monoclonal antibodies, despite their importance in cancer research, have not offered the expected curative results, so the need for more potent agents was clear in order to completely treat cancer. Further studies using monoclonal antibodies as a treatment were made [11] introducing us to the amazing therapeutic properties of them, especially the selective delivery of cytotoxic agents to tumor cells, creating what is called antibody-drug conjugates (ADCs) as a solution to increase the therapeutic index of a cytotoxic chemical agent [11, 12]. Although these HER2-targeting therapies have improved the overall survival rate, many more cases are still not affected by these treatments. A large population of them have breast cancer which does not overexpress HER2 receptors, those are clinically categorized as “HER2-negative.” The word “negative” does not mean that the tumor does not express any HER2 receptors; it actually means that the amount of the receptors is not enough for the anti-HER2 antibodies to be used as a treatment [13]. If the tumor also does not express hormone receptors (HR), then it is called triple negative breast cancer (TNBC). In this review, we are going to describe ADCs generally and ADCs used in managing breast cancers specifically.

## 2. Antibody-Drug Conjugates (ADCs)

ADCs are a new class of protein-based therapeutic agents which bring together the targeting capabilities, high selectivity, and stability of mAbs with the cancer-killing potential of highly potent payloads to increase precise drug delivery in cancer cells, while sparing healthy tissues and/or cells from chemotherapeutic damage. This ability of discrimination between normal and cancerous cells would not have been achieved without decades of development of mAbs [9, 14–18].

## 3. Antibody-Drug Conjugates (ADCs) Structure

**Figure 1:**
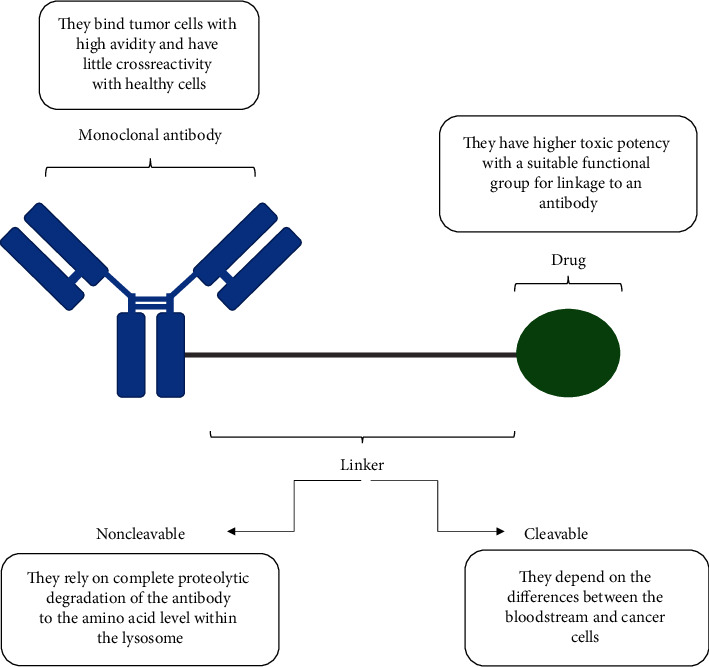
Structure of an antibody-drug conjugate [19].

In order to achieve the desired results, each ADC must contain three parts (Figure 1) [19]:

Monoclonal antibody: it binds the ADC to a specific tumor cell surface protein [19]. The antibody should bind tumor cells with high avidity and have little crossreactivity with healthy cells so that it does not affect them. All the antibodies developed or currently in clinical trials are immunoglobulin G (IgG); taking advantage of that, they contain multiple native sites for conjugation and can be modified for additional reactive sites [20, 21]. Most of the ADCs are built on IgG1 scaffolds because the antibody-dependent cell mediated cytotoxicity (ADCC) and complement-dependent cytotoxicity (CDC) are much stronger in IgG1 and IgG3 than IgG2 and IgG4 [22, 23].

Linker: it is a chemical spacer between the cytotoxic drug and the monoclonal body. It is usually stable in the circulation, but most of the linkers are easily displaced inside the cells. However, if the linker stays stable inside the cell, it requires degradation to release the drug. The linker must allow the release of the drug in its active form within or close enough to the target cells, because early release of drugs in the circulation can result in systemic toxicity and a lower therapeutic index [24, 25]. There are 2 types of linkers: cleavable and noncleavable, both of them are used in the ADCs developed or currently in clinical trials [26]. Cleavable linkers depend on the differences between conditions in the bloodstream and the cytoplasmic conditions within cancer cells (low pH, proteolytic cleavage, and high glutathione concentrations). Depending on their response to intracellular conditions, there are three types of cleavable linkers: hydrazone, disulfide, and peptide linkers [16, 27]. On the other hand, noncleavable linkers release the drug after internalization in the target cell [16, 27]; they rely on complete proteolytic degradation of the antibody to the amino acid level within the lysosome [27], that means they require appropriate internalization and degradation inside the cell to be active. The most common example of noncleavable linkers is the thioether linker.

Cytotoxic drug: cytotoxic compounds are divided into two main categories: microtubule inhibitors and DNA-damaging agents. There are also other small molecules under investigation [27, 28]. All the cytotoxic compounds used in the ADC structure must have higher toxic potency compared with standard chemotherapeutic agents, be able to kill cancer cells by induction of apoptosis, have a suitable functional group for linkage to an antibody, and be soluble in aqueous solutions to enable the reaction with antibodies [24, 29, 30].

## 4. Development of Antibody-Drug Conjugates

There are three generations of ADCs: first, second, and third.  Table 1shows the main differences between them.

## 5. Mechanism of Action of HER2-Directed ADCs [34]

### 5.1. Classical Mode of Action

The monoclonal anti-HER2 binds to the HER2 expressed on the cells of the tumor and gets internalized by endocytosis. The proteases in the lysosomes cleavage the linker, releasing the payload and starting the cytotoxic effects.

### 5.2. Bystander Killing Effect

This effect happens when the ADC is designed that the antibody releases the payload before internalization so that the surrounding cells get affected by its cytotoxic effects even if they do not express the receptor.

## 6. Antibody-Drug Conjugates (ADCs) Targeting HER2+ Receptors

### 6.1. A166 [35]

1. Monoclonal antibody: a human epidermal growth factor receptor 2 (EGFR2; HER2; ERBB2) targeting monoclonal antibody.
2. Payload: an undisclosed cytotoxic agent with potential antineoplastic activity.
3. Development status: first in human phase I/II.

### 6.2. ADCT-502 [36]

1. Monoclonal antibody: an engineered version of the humanized monoclonal antihuman epidermal growth factor receptor 2 (HER2) immunoglobulin G1 (IgG1) trastuzumab.
2. Linker: cathepsin B-cleavable valine-alanine linker.
3. Payload: DNA cross-linking pyrrolobenzodiazepine (PBD) dimer-based drug tesirine.
4. Development status [37]: phase I.

### 6.3. ALT-P7 [38]

1. Monoclonal antibody: trastuzumab biobetter HM2.
2. Payload: monomethyl auristatin E (MMAE).
3. Development status [34]: phase I.

### 6.4. Anti-HER2-vc0101 [39]

1. Monoclonal antibody: a human epidermal growth factor receptor 2 (HER2) site-specifically targeting monoclonal antibody.
2. Linker: cleavable valine-citrulline- linker.
3. Payload: an analog of dolastatin 10, auristatin-0101.

### 6.5. ARX788 [40]

1. Monoclonal antibody: a human epidermal growth factor receptor 2 (EGFR2; HER2) site-specifically targeting monoclonal antibody.
2. Linker: para-acetyl-phenylalanine (pAcF linked to a nonnatural amino acid linker.
3. Payload: auristatin analog and potent microtubule inhibitor monomethyl auristatin F (MMAF).
4. Development status [41]: preclinical studies, phase I.

### 6.6. BAT8001 [42]

1. Monoclonal antibody: a human epidermal growth factor receptor 2 (EGFR2; HER2; ErbB2) targeting monoclonal antibody.
2. Payload: undisclosed maytansine derivative.
3. Development status [43]: BAT8001 is in phase III clinical evaluation as a treatment of HER2-positivemetastatic breast cancer that is treated previously with trastuzumab..

### 6.7. DHES0815A [44]

1. Monoclonal antibody: a monoclonal antibody targeting human epidermal growth factor receptor 2 (ERBB2; EGFR2; HER2).
2. Payload: a DNA minor groove cross-linking agent pyrrolo[2, 1-c][1, 4]benzodiazepine monoamide (PBD-MA).
3. Development status [34]: first-in-human (FIH), phase I, open-label, multicenter, dose-escalation study.

### 6.8. Disitamab Vedotin [45]

1. Monoclonal antibody: a monoclonal antibody targeting human epidermal growth factor receptor 2 (ERBB2; EGFR2; HER2).
2. Linker: a cleavable maleimidocaproyl-valyl-citrullinyl-p-aminobenzyloxycarbonyl (mc-val-cit-PABC) type linker.
3. Payload: monomethyl auristatin E (MMAE).
4. Development status: phase I and phase II .

### 6.9. LCB14-0110 [46]

1. Monoclonal antibody: a monoclonal antibody against human epidermal growth factor receptor 2 (HER2) site-specifically.
2. Payload: monomethyl auristatin F (MMAF).
3. Development status: LegoChemistry™ and ADC platform technology ConjuAll™.

### 6.10. Hertuzumab Vedotin [47]

1. Monoclonal antibody: hertuzumab.
2. Payload: monomethyl auristatin E (MMAE).
3. Development status: phase I and phase II.

### 6.11. MEDI4276 [48]

1. Monoclonal antibody: a bispecific antibody against the extracellular domain of human epidermal growth factor receptor 2 (HER2; ERBB2) comprised of the single-chain variable fragment (scFv) of the anti-HER2 monoclonal antibody trastuzumab, fused to the heavy chains of the anti-HER2 monoclonal antibody 39S.
2. Payload: tubulysin
3. Development status [49]: phase I.

### 6.12. MI130004 [50]

1. Monoclonal antibody: trastuzumab.
2. Linker [51]: it has a maleimide group to facilitate conjugation to Cys residues.
3. Payload: PM050489.
4. Development status [51]: preclinical.

### 6.13. MM-302 [52]

1. Monoclonal antibody: a monoclonal antibody against the human epidermal growth factor receptor 2 (HER2).
2. Payload: the antineoplastic anthracycline antibiotic doxorubicin encapsulated within liposomes.
3. Development status [53]: phase II.

### 6.14. Trastuzumab Deruxtecan [54]

1. Monoclonal antibody: a monoclonal antibody targeting human epidermal growth factor receptor 2 (ERBB2; EGFR2; HER2).
2. Linker: a tetrapeptide linker, Gly-Phe-Leu-Gly (GFLG).
3. Payload: deruxtecan, a derivative of the camptothecin analog exatecan (DXd; DX-8951 derivative); a DNA topoisomerase 1 (topoisomerase I; Top1) inhibitor, with antineoplastic activity.
4. Development status [55]: FDA approval based on the results of the registrational phase II trial DESTINY-Breast01.

### 6.15. Trastuzumab Duocarmazine [56]

1. Monoclonal antibody: trastuzumab, a monoclonal antibody targeting epidermal growth factor receptor 2 (HER2).
2. Linker [57]: a cleavable linker N-[2-(2maleimidoethoxy)ethoxycarbonyl]-L-valyl-L-citrullinyl-p-aminobenzyloxycarbonyl-N-[2-(2-hydroxyethoxy)ethyl]-N-[2-(methylamino)ethyl]carbamoyl.
3. Payload: the duocarmycin prodrug.
4. Development status [57]: phase I, phase II, and phase III.

### 6.16. Trastuzumab Emtansine [58]

1. Monoclonal antibody: trastuzumab.
2. Linker [59]: noncleavable succinimidyl-4-(N-maleimidomethyl) cyclohexane-1-carboxylate (SMCC) linker
3. Payload: the microtubule-inhibitory agent DM1.
4. Development status [59]: approved 2013 and approved 2019.

### 6.17. XMT-1522 [60]

1. Monoclonal antibody: HT-19, a monoclonal antibody directed against the human epidermal growth factor receptor 2 (ERBB2; HER2) that binds to domain IV of HER2 to an epitope that is distinct from the trastuzumab-binding site
2. Payload: proprietary auristatin-derived payload molecules.
3. Development status [61]: in January 2019, phase I studies for breast cancer (late-stage disease, metastatic disease), gastric cancer (late-stage disease), and nonsmall cell lung cancer (late-stage disease) are discontinued (United States)

## 7. Antibody-Drug Conjugates (ADCs) Targeting Triple Negative Breast Cancer (TNBC)

### 7.1. Sacituzumab Govitecan [62]

1. Monoclonal antibody: sactizumab, an anti-Trop-2 humanized antibody.
2. Payload: the topoisomerase-I inhibitor SN-38.
3. Development status [63]: phase I/II.

### 7.2. Ladiratuzumab Vedotin [64]

1. Monoclonal antibody: the zinc transporter LIV-1 targeting humanized antibody.
2. Payload: monomethyl auristatin E (MMAE).
3. Development status: phase I.

### 7.3. AVID100 [65]

1. Monoclonal antibody: anti-EGFR antibody.
2. Payload: DM1 (derivative of maytansine).
3. Development status: phase I.

### 7.4. U3-1402 [66]

1. Monoclonal antibody: an anti-HER3 antibody.
2. Payload: a topoisomerase I inhibitor exatecan derivative (DXd).
3. Development Status: phase I/II.

### 7.5. CAB-ROR2-ADC [67]

1. Monoclonal antibody: receptor tyrosine kinase-like orphan receptor 2 (ROR2) targeting antibody.
2. Payload: an undisclosed payload.
3. Development status: phase I/II.

### 7.6. Anti-CA6-DM4 Immunoconjugate [68]

1. Monoclonal antibody: a humanized DS6 antibody directed against tumor-associated sialoglycotope CA6.
2. Payload: the maytansinoid DM4.
3. Development status: phase I.

## 8. Conclusion

Breast cancer has become the most common cancer in the world; a lot of treatment methods and technologies were used in order to control it, but all of them did not achieve the required goal, until the invention of the antibody-drug conjugates. The concept of targeted delivery of anticancer drugs helped the oncologists to improve the tumor selectivity of anticancer drugs and to lower their systemic toxicity. Meaning that these drugs could be administered at higher doses, providing better therapeutic benefit to their patients. The tumor selectivity of antibodies offered a chance to achieve this goal by using them as guide for the drug towards the tumor. This seemingly simple concept had great attention from researchers at academic institutions and in the pharmaceutical industry. The current breed of ADCs uses antibodies that are humanized, not immunogenic, and linkers that are designed to be stable in circulation, but are cleaved upon delivery into a cell. The recent FDA approvals of new ADCs have generated tremendous excitement. There are a lot of ADCs currently in clinical evaluation and almost every major pharmaceutical company has embraced this technology. There is active research by medicinal chemists to develop new linkers and discover new potent effector molecules suitable for use in ADCs, while biologists have focused on identifying cell-surface targets suitable for antibody development.

## Figures and Tables

**Table 1:** Main characteristics of the ADC generations.

First-generation [29, 31]	Second-generation [29, 32]	Third-generation [33]
(1) Anticancer drugs were coupled through noncleavable linkers to murine mAbs. (2) Evaluations showed that they were only moderately potent and less active than the parent drugs. (3) Examples: KS1/4-methotrexate and BR96-doxorubicin.	(1) Huge improvements in mAbs technology were made, increasing selective binding to tumor cells and reducing crossreactivity with healthy cells. Payloads with smaller molecules were also discovered. (2) Examples: brentuximab vedotin, ado-trastuzumab emtansine, and inotuzumab ozogamicin.	(1) Site-specific conjugation was developed, improving the therapeutic index, stability, and potency. (2) Examples: MEDI4276, vadastuximab talirine, and IMGN779.
